# A case report: a giant cardiac atypical lipoma associated with pericardium and right atrium

**DOI:** 10.1186/s12872-019-1221-1

**Published:** 2019-11-06

**Authors:** Xin Wang, Xiaona Yu, Weidong Ren, Dongyu Li

**Affiliations:** 1 Department of Ultrasound, Shengjing Hospital of China Medical University, Shenyang, China No.36, Sanhao Street, Heping District, Shenyang City, 110004 China; 2 Department of Cardiac surgery, Shengjing Hospital of China Medical University, Shenyang, China

**Keywords:** Atypical lipoma, Echocardiography, Cardiac tumor

## Abstract

**Background:**

Among primary cardiac tumors, atypical lipoma is very rare. In particular, cases with lipomas in both the pericardium and the atria are even rare.

**Case presentation:**

We report the case of a 49-year-old male patient presented to our department because of chest pain. Echocardiography revealed two large masses in both the pericardium and the right atrium. Then the tumors were completely resected and the histopathological examination revealed atypical lipoma. The patient recovered well without any complication and discharged from hospital.

**Conclusions:**

We report a very rare case of a huge atypical lipomas located on the pericardium and right atrium. These tumors were easily detected by echocardiography and final diagnosed after surgical resection and pathological examination.

## Background

Primary cardiac tumor are not conmen and the incidence range of 0.0017–0.28% as reported in autopsy studies [1]. One quarters of cardiac tumors can be categorized as malignant tumor on histology. The most common malignant tumors of the heart is angiosarcoma whereas atypical lipomas represent < 1% [2, 3]. Cardiac atypical lipomas are usually found incidentally because of asymptomatic, and in most cases require treatment or surgical intervention. We report a case of a 49-year-old male patient was referred to our hospital because of chest pain. Echocardiography revealed a giant mass in the pericardial cavity, and another mass in the right atrium.

## Case presentation

**Fig. 1:**
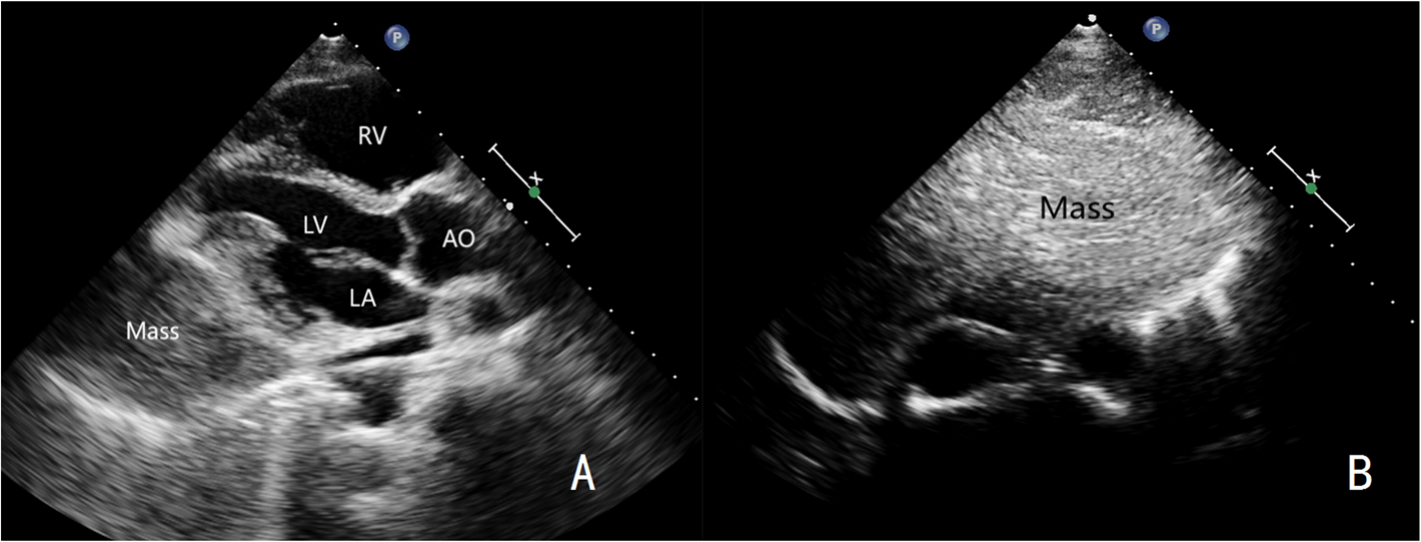
**a** Left ventricular long axis view shows a large mass in the pericardium. **b** Subxiphoid view shows a giant mass in the pericardium. LA, left atrium; LV, left ventricle; RV, right ventricle; AO: aorta

**Fig. 2:**
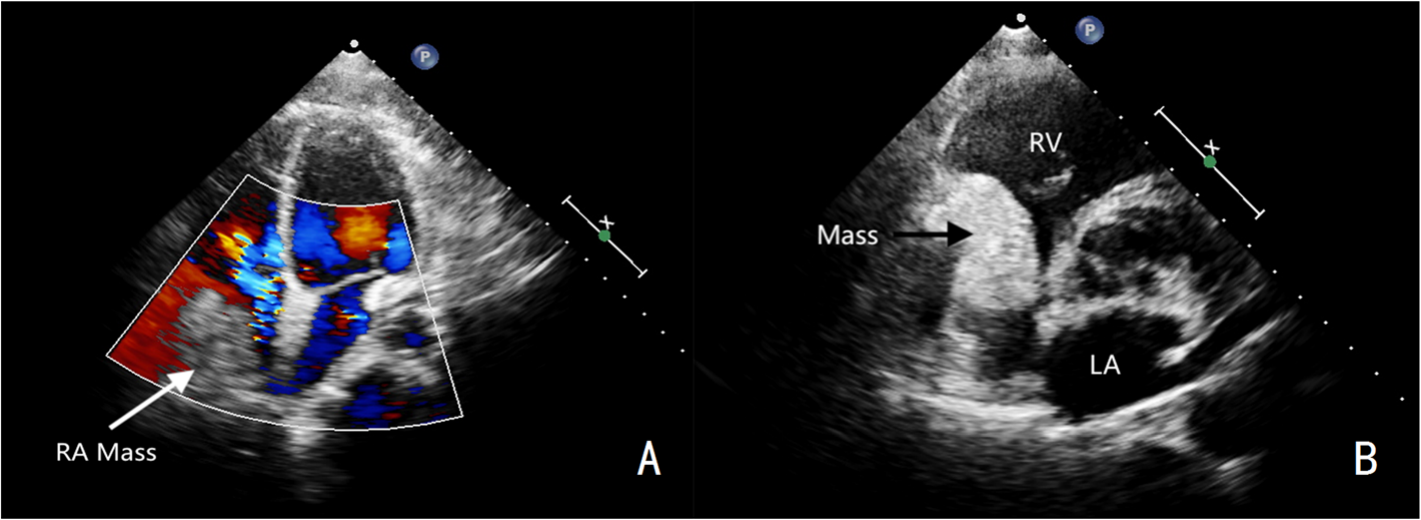
**a** The apical four chamber view demonstrates the right atrial mass (arrow), there is no blood flow signal within the mass. **b** During diastolic period, the mass (arrow) moves toward the tricuspid valve orifice, but does not enter the right ventricle. LA, left atrium; RV, right ventricle

**Fig. 3:**
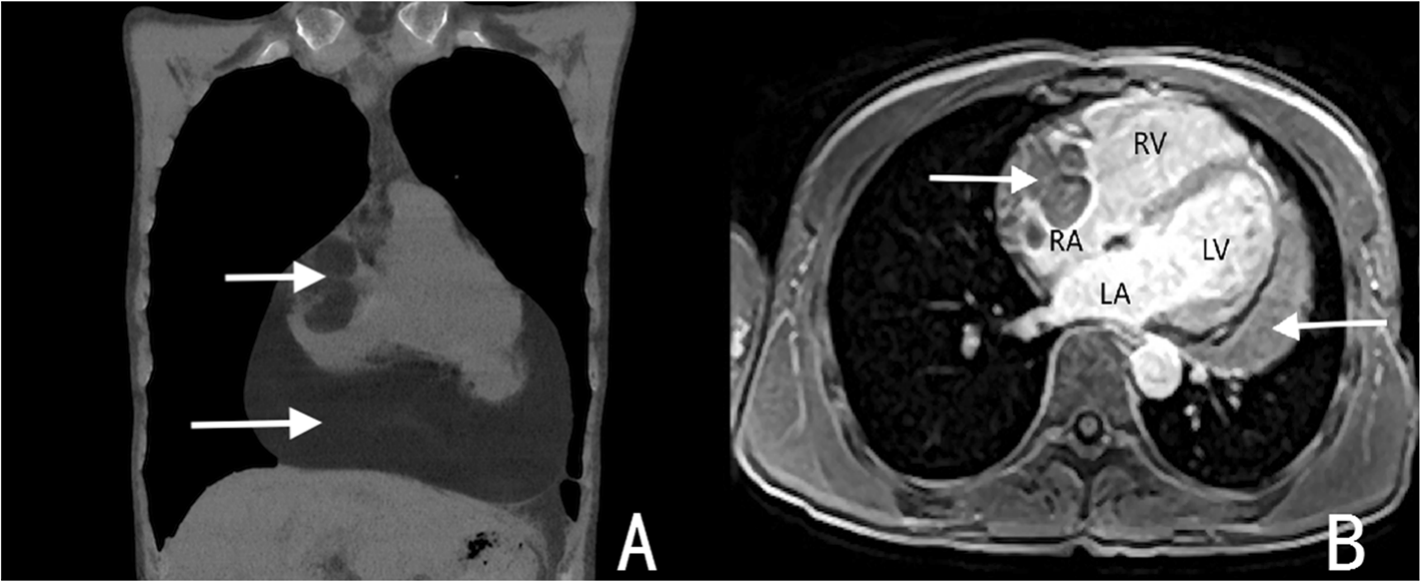
Magnetic resonance imaging examination. **a** In the coronal section, shows the pericardium and atrial mass (arrow) **b** In the long axis four chamber view, shows the pericardium and atrial mass (arrow)

**Fig. 4:**
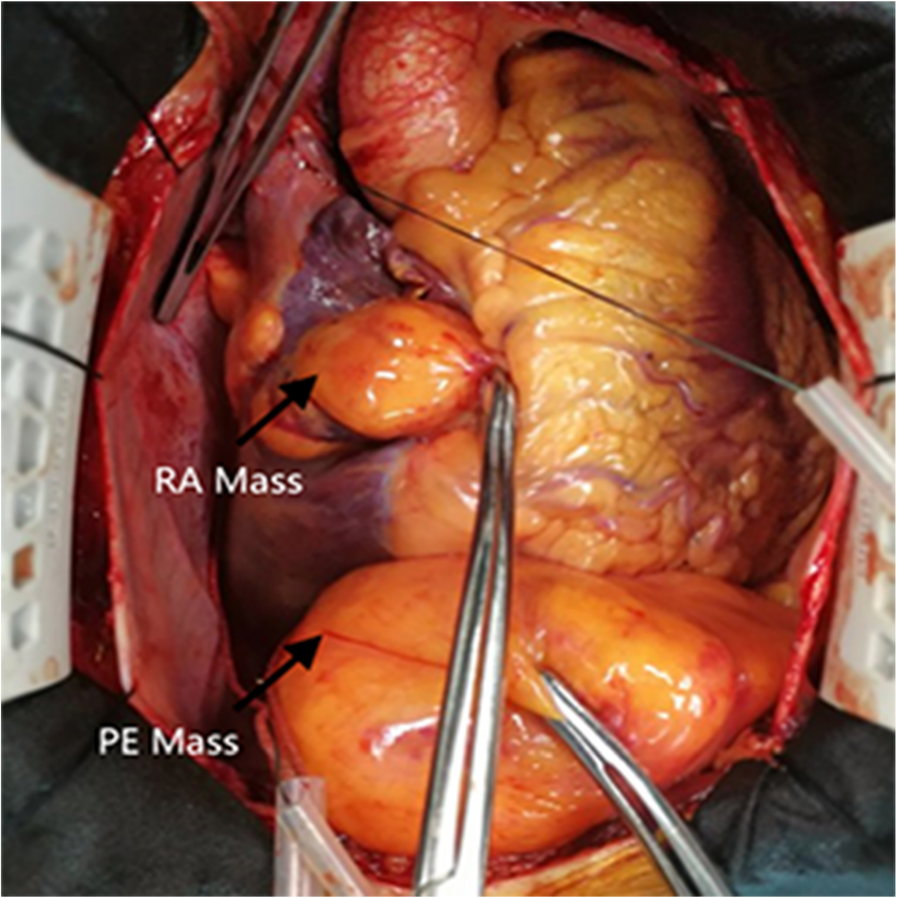
An intraoperative photograph shows a giant mass which locates in the pericardial cavity, another yellowish fatty mass is revealed in the right atrium

**Fig. 5:**
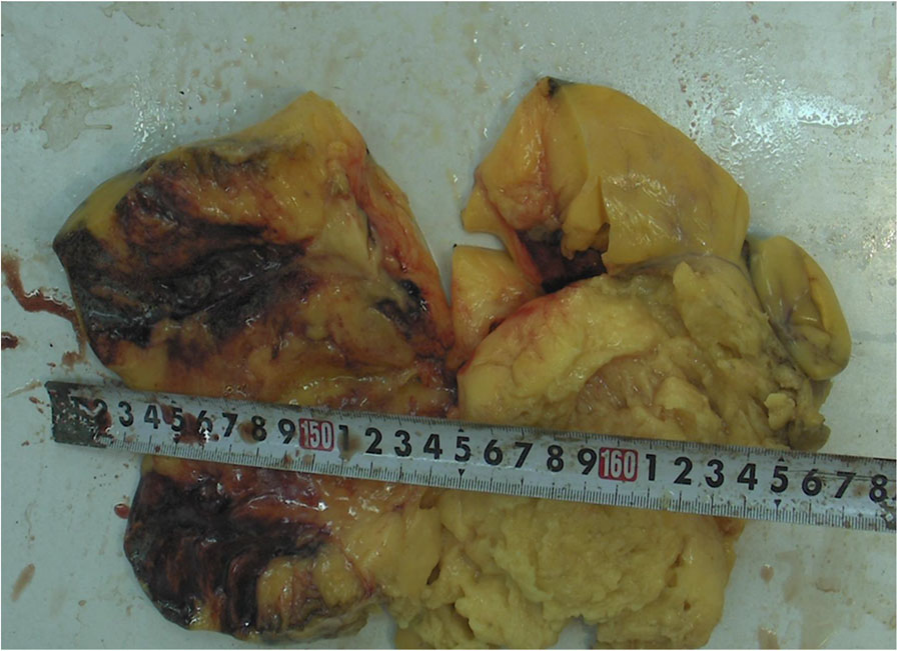
Macroscopic inspection reveals several multilobulated fatty mass

**Fig. 6:**
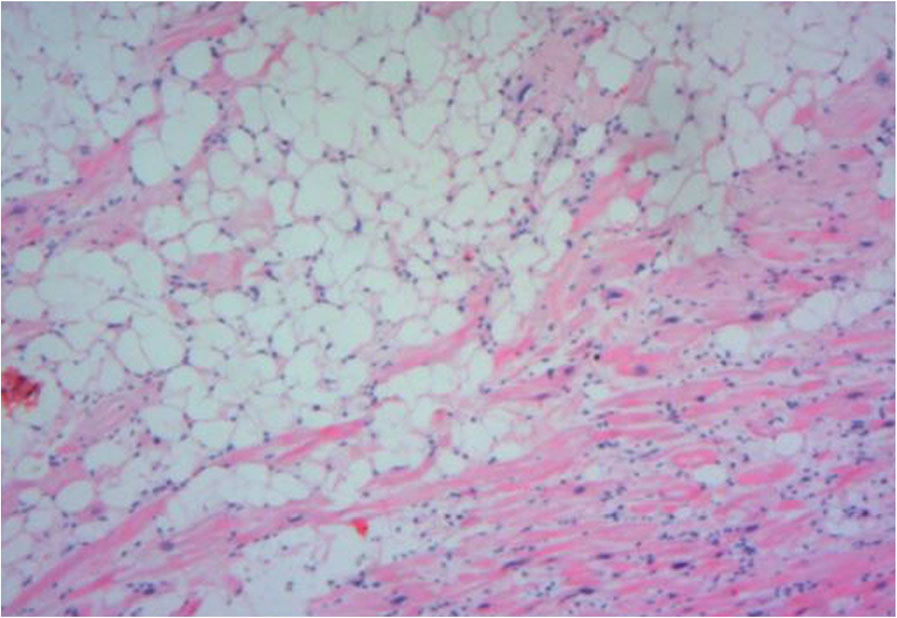
Pathological examination shows a sheet-like arrangement of adipocyte. The nuclei of local lesion cells slightly enlarge (H&E staining; magnification, × 20). H&E, haematoxylin and eosin

A 49-year-old male was admitted to our hospital because of chest pain over 20 days. Physical examination and electrocardiography were normal. Transthoracic echocardiography showed a giant solid echogenic mass in the pericardial cavity (Fig. 1). The size of the mass was about 16.2 × 10.5 cm. Another echogenic mass measuring 4.8 × 3.1 cm was observed in the right atrium (Fig. 2). The right atrial mass has a significant degree of activity with the cardiac cycle. During diastole period, the mass moved toward the tricuspid valve orifice, but did not enter the right ventricle. The tricuspid valve orifice blood flow was not obstructed. There was no blood flow signal within the two masses. These findings were confirmed by chest magnetic resonance imaging (Fig. 3). At surgery, the pericardium was opened, a giant mass measuring 15cmx12cmx8cm was found in the pericardial cavity, another yellowish fatty mass was revealed in the right atrium (Fig. 4). Then the tumors were completely resected and the resected material was sent for pathological study. Macroscopic inspection revealed several multilobulated fatty masses (Fig. 5). Histologic examination showed adipocytes and confirmed the diagnosis of atypical lipoma (Fig. 6). The patient’s postoperative course was uneventfully and he recovered quickly.

## Discussion and conclusions

Liposarcoma–a mesenchymal malignant tumor that contains lipoblasts–is the most common soft tissue sarcoma and accounts for 20% of all mesenchymal tumors [4]. The tumor occurs most frequently in the limbs and retroperitoneum and rarely originates in the heart [5]. Peak incidence is between 40 and 60 years old, and there is a slight male predominance [6]. Liposarcoma of the heart is an extremely rare sarcoma, with less than 35 cases reported in the literature according to literature [7]. To our best knowledge, this is the first reported case of simultaneous atypical lipoma in the right atrium and pericardium.

Liposarcomas can be divided into four subtypes: Well-differentiated liposarcoma (or atypical lipoma), myxoid/round cell liposarcoma, pleomorphic liposarcoma, and dedifferentiated liposarcoma. Atypical lipoma was the most common subtype (50%), which had been observed in the atrium and the pericardium. The tumor usually grows slowly, insidious and remain asymptomatic until reach a giant size. Atypical lipomas can be asymptomatic or symptomatic depending on their size and location within the heart. They can invade adjacent organs, so they are more likely to be symptomatic than benign lipomas. Atypical lipomas have a mild biological behavior and do not generally metastasis, but there is a possibility of focal recurrence.

Although pathological diagnosis serves as a gold standard of diagnosis of atypical lipoma, echocardiography is still essential. Echocardiography is an ideal and important diagnostic modality since it can provide information of the tumor on the size, location, boundary, modality, amount blood flow and so on. Although echocardiography offers a simple and non-invasive approach, it cannot conclusively differentiate between atypical lipomas and other primary tumors of the heart. Computed tomography or magnetic resonance imaging may be of additional value.

There are several diseases should be differentiated from atrial atypical lipomas, namely myxoma, thrombus, benign lipoma. Atrial myxoma is the most frequent primary cardiac tumor in adults. It usually arises in the area of the fossa ovalis, grows on pedicles, has the manner of soft and polyps. The tumor may move towards the ventricle during diastole. Atrial thrombus is usually associated with a history of atrial fibrillation or rheumatic heart disease, and ventricular thrombosis usually occurs on the basis of abnormal wall motion. The base of the thrombus is wide and can have activity. As time progresses, the echo of the thrombus can gradually change. The size of the thrombus may change after anticoagulant therapy. Cardiac benign lipomas can originate either from the subendocardium (50%), subpericardium (25%), or myocardium (25%) and maybe located more frequently in the left ventricle or right atrium [8]. Cardiac benign lipomas are most often detected incidentally and do not have specific ultrasound findings. The lipoma demonstrated typical loss of signal on fat suppressed magnetic resonance imaging sequence techniques.

Pericardial atypical lipomas may confuse with malignant mesothelioma, pericardial cyst, hemangiomas. The ultrasound manifestations of malignant mesothelioma usually include pericardial effusion, pericardial thickening, pericardial cavity mass. Pericardial cysts are usually congenial lesion or acquired [9]. Echocardiography showed an isolated, unilocular, smooth-walled cystic mass adjacent to the heart. There was no communicating tract between mass and heart cavity, and no blood flow signal within the mass. Cardiac hemangiomas are extremely rare and account for approximately 2% of all primary resected cardiac tumors. They can locate in atrium、ventricle、pericardium and endocardium [10]. Echocardiography usually presents as a hyperechoic mass with a well-defined boundary, and myocardial contrast echocardiography can demonstrate the vascularity of the mass.

In conclusion, we report a very rare case of a huge atypical lipomas located on the pericardium and right atrium. These tumors were easily detected by echocardiography and final diagnosed after surgical resection and pathological examination. He is under follow-up for the possibility of recurrence.

## Data Availability

The datasets used in the case are available from the corresponding author upon reasonable request.
